# Extracellular Vesicles and Epigenetic Modifications Are Hallmarks of Melanoma Progression

**DOI:** 10.3390/ijms21010052

**Published:** 2019-12-20

**Authors:** Francesco Mannavola, Stella D’Oronzo, Mauro Cives, Luigia Stefania Stucci, Girolamo Ranieri, Franco Silvestris, Marco Tucci

**Affiliations:** 1 Department of Biomedical Sciences and Clinical Oncology, University of Bari, ‘Aldo Moro’, 70121 Bari, Italy; francesco.mannavola@gmail.com (F.M.); ester86d@gmail.com (S.D.); mauro.civ@tiscali.it (M.C.); stuccistefania@gmail.com (L.S.S.); francesco.silvestris@uniba.it (F.S.); 2 National Cancer Research Center, Istituto Tumori ‘Giovanni Paolo II’, 70121 Bari, Italy; giroran@tiscalinet.it

**Keywords:** melanoma, extracellular vesicles, epigenetic modifications, miRNAs

## Abstract

Cutaneous melanoma shows a high metastatic potential based on its ability to overcome the immune system’s control. The mechanisms activated for these functions vary extremely and are also represented by the production of a number of extracellular vesicles including exosomes. Other vesicles showing a potential role in the melanoma progression include oncosomes and melanosomes and the majority of them mediate tumor processes including angiogenesis, immune regulation, and modifications of the micro-environment. Moreover, a number of epigenetic modifications have been described in melanoma and abundant production of altered microRNAs (mi-RNAs), non-coding RNAs, histones, and abnormal DNA methylation have been associated with different phases of melanoma progression. In addition, exosomes, miRNAs, and other molecular factors have been used as potential biomarkers reflecting disease evolution while others have been suggested to be potential druggable molecules for therapeutic application.

## 1. Introduction

Cutaneous melanoma (CM) develops from the malignant transformation of neural crest-derived melanocytes and its incidence has progressively increased worldwide. In recent decades, remarkable progress has been made in understanding the molecular landscape of CM and revealing a crucial pathogenic role of the *BRAF* mutation and aberrant activation of the mitogen-activated protein kinase (MAPK) pathway as major mechanisms driving melanomagenesis [1]. Several additional alterations, however, co-operate in melanoma progression, including the intercellular trafficking of extracellular vesicles (EVs) and epigenetic rearrangements [2].

The spreading of melanoma cells from the primary site to metastatic tissues is regulated by a complex sequence of events mostly activated within the tumor microenvironment as a consequence of the interplay between tumor and immune cells as well as other components of the stroma. Apart from the direct cell-to-cell contact and the indirect effect exerted by soluble factors and cytokines, recent studies also suggested an alternative way for intercellular communication based on the continuous shedding of EVs into the tumor bed [3]. These vesicles are delimited by a lipid bilayer showing variable sizes and endosomal/cytosolic derivation, whose active cargo reveals specific functions in modulating intracellular signalling and gene expression. Particularly, exosomes (Exo) are nano-sized EVs that have been largely investigated in melanoma [4]. Exosomes show the ability to drive a number of specialized functions implicated in the control of proliferation, epithelial-mesenchymal transition (EMT), immune-evasion, and pre-metastatic niche formation [5,6].

In this complex scenario, the progressive accumulation of molecular defects along melanomagenesis also depends on the epigenetic regulation of several oncogenes and oncosuppressor genes, mostly ruled by DNA methylation, histone, and chromatin modifications as well as non-coding RNA deregulation [7,8,9]. The understanding of these processes paves the way for alternative diagnostic tools and novel therapeutic approaches, which are also favored by the overwhelming improvements of technology and bioinformatics.

In this paper, we review recent studies on the role of EVs and epigenetic defects in promoting melanoma development and propagation of malignant cells toward metastatic sites.

## 2. Extracellular Vesicles and Melanoma

### 2.1. Classification and Biogenesis

**Figure 1:**
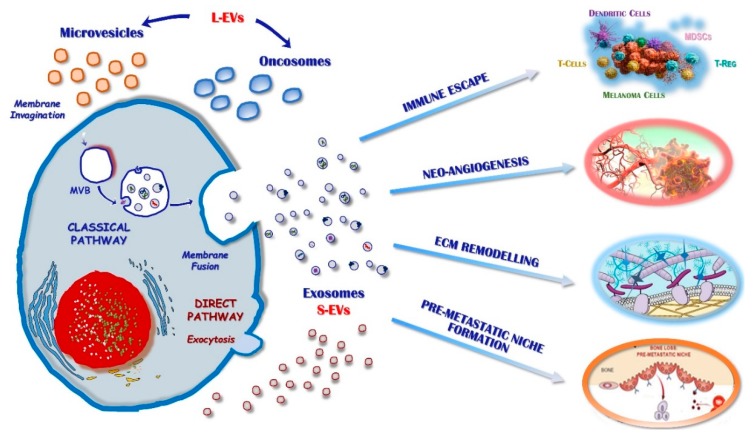
Extracellular vesicles (EVs) can be defined as large (L) or small (S) based on their diameter and include: micro-vesicles (>0.5 µm), apoptotic bodies (0.8–5 µm), oncosomes (1–10 µm), and exosomes (50–130 nm). Large-EVs are generated from the plasma membrane by direct gemmation. Exo may be produced and released through either a “classical” or a “direct” pathway. Exosomes have been shown to support melanoma progression through several mechanisms, including participation in the “immune escape”, neo-angiogenesis, extracellular matrix (ECM) remodelling, and pre-metastatic niches formation.

The International Society for Extracellular Vesicles (ISEV) has recently updated the nomenclature and classification guidelines for EVs, including particles naturally released by cells that are delimited by a lipid bilayer and cannot replicate [10]. These vesicles are further classified in large (L) and small (S)-EVs based on their diameter (Figure 1), respectively, which is larger or smaller than 200 nm. The L-EVs generally include microvesicles or ectosomes (>0.5 µm), apoptotic bodies (0.8–5 µm), and large oncosomes (1–10 µm), while melanosomes (>0.5 µm) are melanin-containing organelles that are specifically produced by melanocytes. On the other hand, S-EVs are mostly represented by Exo (50–130 nm), although other S-EVs have been described including non-membranous nanoparticles named “exomeres” (~35 nm) [11].

L-EVs originate by direct gemmation of the plasma membrane following activation of signal transduction pathways and, therefore, may play a role in intercellular communication in physiological as well as in pathological conditions. Recent studies in prostate cancer revealed the existence of a particular class of very large EVs (1–10 μm), which are known as “oncosomes”, resulting from the shedding of non-apoptotic blebs unique to cancer cells [12]. Although these vesicles have not yet been demonstrated in melanoma, the release of these large vesicles has been mostly correlated with the aberrant expression of several oncoproteins, such as MyrAkt1, HB-EGF, and caveolin-1, which suggests that oncosomes may be a hallmark of cancer cells [13]. Proteins enriched in oncosomes, moreover, include enzymes involved in glucose, glutamine, and amino acid metabolism, which support a direct role of these EVs in the regulation of cancer cell metabolism [14].

Exosomes are particularly enriched in cholesterol and sphingomyelin in their lipid bilayer and express typical EV-related tetraspanins (CD9, CD63, and CD81), proteins derived from the endosomal compartment (e.g., Alix, TSG101, and Heat Shock Protein 90), as well as major histocompatibility complex (MHC) molecules exerting functional antigen presentation to T-cells [11]. Moreover, tumor cell-derived Exo resemble the antigenic repertoire of the producing cells. Exo isolated from metastatic melanoma patients were found enriched in Caveolin-1, Melan-A, gp100, beta-catenin, chondroitin sulfate peptidoglycan-4, and VLA4 [15,16] whose expression is variably correlated with prognosis and metastatic extension [17,18].

The mechanisms implicated in the Exo formation include both classical and direct pathways. The first one produces Exo by inward budding of the plasma membrane and includes the Endosomal Sorting Complex Required for Transport (ESCRT)-dependent and the ESCRT-independent pathways. The activation of ESCRT complexes is required for the formation of multivesicular bodies (MBVs) encapsulating early Exo and their loading with a number of active molecules [19,20]. This machinery produces mature Exo that are released in the extracellular space by fusion of MVBs with the cell membrane [21]. By contrast, the ESCRT-independent pathway involves the ceramide protein that is relevant for the formation of a cone-shaped structure promoting the spontaneous negative curvature of the inner layer of the plasma membrane [22]. An alternative direct pathway depends on the outward budding of the cell plasma membrane and it was previously described for Exo originating from T-cells, which allows the rapid generation and release of nanovesicles into the extracellular space [23].

### 2.2. Role of Exo in Melanoma Progression

Increasing interest in past years has been devoted to understanding the role of Exo in melanoma progression since they are considered relevant intercellular communicators supporting the survival and proliferation of malignant cells as well as the propagation of pro-metastatic signals. Although EVs were originally considered to be cellular waste products, it is now well accepted that they have a key role in intercellular communication, depending on the delivery of active cargos, including proteins, coding-RNAs and non-coding RNAs, and DNA fragments, from donor to distant cells [24,25]. For example, a reciprocal cross-talk mediated by EVs between normal melanocytes and keratinocytes has been described: (i) melanocytes normally release melanosomes, a specific class of large vesicles, which transfer the pigment melanin to nearby keratinocytes in response to ultraviolet exposure [9]. On the other hand, (ii) Exo are normally secreted by keratinocytes and interact with melanocytes, which enhances their melanin synthesis by increasing both the expression and activity of melanosomal proteins [26].

Relatively to melanoma, Exo from highly invasive cells support melanoma progression and metastasis by stimulating the proliferation and invasiveness of nearby tumor cells. In a similar manner, tumor-derived Exo are able to conditionate the organotropism of melanoma cells as recently reported by our group using an in vitro model of bone metastasis [8,27]. Moreover, Exo facilitate the EMT of melanoma cells consisting of the acquisition of the mesenchymal phenotype and pro-invasive behavior by up-regulating Let7a, Let7i, and miR-191, which, in turn, down-regulate E-cadherin and activate the expression of vimentin, ZEB2, and SNAIL2 [28]. In addition, Exo may also vehiculate pro-angiogenic molecules that promote the neo-angiogenesis and extracellular matrix (ECM) remodelling, such as interleukin (IL)-6, vascular endothelial factor (VEGF), and different metalloproteinase (MMPs) [29]. The formation of a new vascular network and the remodelling of the extracellular space are crucial for the detachment of melanoma cells from the primary site, which represents the first steps of the metastatic cascade.

Lunavat et al. have recently demonstrated that the inhibition of BRAFV600E melanoma cells with vemurafenib is associated with an increase in the release of EVs, which result in specific RNA [30]. The authors have correlated these adaptations with a mechanism of acquired resistance to BRAF inhibitors and suggested that intercellular communication mediated by such EVs is likely to be important for melanoma progression. Similarly, another group found that Exo produced by melanoma cells pre-treated with alkylating drugs can induce in vivo chemoresistance through the up-regulation of genes involved in DNA damage repair and cell survival, which promotes tumor growth after treatment [31]. Therefore, these results indicate that treatment of melanoma cells may cause significant changes in the protein and RNA cargo of the EVs that they excrete, which contributes to drug resistance and melanoma progression.

Exosomes from melanoma cells also take part in the immune-escape, either directly inhibiting immune effector cells or indirectly stimulating regulatory cells. The direct modulation of immune cells largely occurs in response to melanoma Exo-related inhibitory or pro-apoptotic signals and are mostly related to the exosomal expression of PD-L1 [32], while the indirect way involves the up-regulation of PD-1 by tumor accessory cells, such as mesenchymal stem cells (MSCs), which engulf the tumor microenvironment and restrain T-cell activation [33]. Moreover, Bland et al. have recently discovered an alternative mechanism of melanoma derived-Exo, which affect the epigenetic landscape and mitochondrial respiration of cytotoxic T-cells. This results in their reduced activation [34]. Studies in different types of cancer also demonstrate that tumor-derived EVs restrain the expansion and differentiation of myeloid-derived suppressor cells (MDSCs) and T-regulatory cells (Treg) [35,36] as well as reduce the cytotoxic activity of natural killer (NK) cells by down-regulating NKG2D, NKp30, NKP46, and NKG2C receptors [37].

Lastly, Exo from melanoma cells migrate toward distant tissues to prepare the pre-metastatic niche. In this regard, Exo enter the lymphatic vessels and are captured into the lymph nodes where it induces the expression of pro-angiogenic factors, including tumor necrosis factor (TNF)-α, VEGF, hypoxia-inducible factor (HIF)-1, and a urokinase plasminogen activator, which enhances the subsequent trapping and growth of melanoma cells [38,39]. Peinado et al. demonstrated that Exo may also prime bone-marrow-derived cells (BMDCs) to acquire a pro-vasculogenic phenotype through the horizontal transfer of tyrosine kinase receptor MET, which enhances recruitment into future metastatic sites to establish a suitable milieu for efficient homing and out-growth of circulating melanoma cells [40]. Proteomic analyses of Exo isolated from different tumors revealed that distinct patterns of integrin expression correlate with specific organotropism like up-regulation of α_6_β_4_ and α_6_β_1_, which are mostly linked to lung tropism, while α_v_β_5_ is mostly linked to the liver [41].

## 3. Epigenetic Modifications and Melanoma Progression

Epigenetic classically refers to the study of inherited gene expression modifications without alterations of the genotype. It has been previously defined as a “stably heritable phenotype resulting from changes in a chromosome without modifications of the DNA sequence” [42]. Alteration of the epigenetic control of gene expression has been linked with a variety of pathological conditions, including neurological, metabolic, and autoimmune disorders as well as cancer. Besides the well-known role of driver mutations along the cancerogenesis, several epigenetic mechanisms co-participate in the neoplastic cellular transformation, primarily depending on the chromatin structure reorganization with consequent aberrant or repressed gene expression [43].

**Figure 2:**
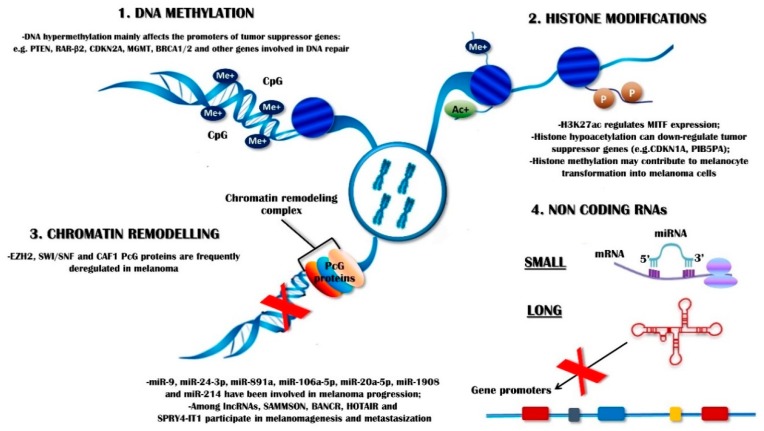
Several epigenetic aberrations have been shown to contribute to melanoma progression and depend on DNA methylation (1), histone post-transcriptional modifications (2), chromatin remodelling (3), and non-coding RNAs rearrangements (4). Red cross: gene transcription down-regulation through inactivation of gene promoters (coloured boxes).

Relatively to melanoma, its complex genetic landscape was partly defined by extensive data from The Cancer Genome Atlas (TCGA) project that demonstrated the high occurrence of somatic mutations and the prevalent activation of the mitogen-activated protein kinase (MAPK) cascade [44]. Moreover, recent technological progress was based on the use of high-throughput methods for DNA-sequencing that allowed us to globally study the details of the epigenome, which revealed aberrant DNA methylation in melanoma at specific gene promoters, histone modifications, alterations in chromatin regulators, and miRNA rearrangement [45,46]. Therefore, these epigenetic aberrations may contribute to the neoplastic transformation of melanocyte, and support the immune-escape of melanoma, the development of metastasis, and both innate and acquired drug resistance (Figure 2).

### 3.1. DNA Methylation

Methylation of DNA is the most investigated mechanism of epigenetic regulation and refers to the addition of a methyl group to the 5-carbon position of cytosine, generally occurring in specific regions of DNA (CpG sites), where a cytosine nucleotide is followed by a guanine in a repeated tandem of 300–3000 base pairs (CpG islands). The CpG islands are located throughout the genome and cover approximately 40–60% of gene promoters, whose hypermethylation status functions as a down-regulator of gene transcription [47].

The hypermethylation of specific tumor suppressor gene promoters has been described in melanoma and prevalently affects those genes involved in the cell cycle regulation, intracellular signalling, and apoptosis, including *phosphatase and tensin homolog* (*PTEN*), *retinoic acid receptor* (*RAR*)-*β2*, and *cyclin-dependent kinase Inhibitor 2A* (*CDKN2A*) [46,48]. This modality of transcriptional inactivation has also been seen in different tumors involving other DNA repair systems, including genes regulating the nucleotide excision repair (NER) [49], mismatch excision repair (MMR) [50], and homologous recombination (BRACA1/2) [51]. Epigenetic inactivation through gene promoter hypermethylation of another important enzyme repairing DNA alkylation damage, namely O^6^-methylguanine-DNA methyltransferase (MGMT), has been previously demonstrated in metastatic specimens of cutaneous melanoma, as compared to primary tumors, and was present in approximately one-third of cases [52].

A minority of melanomas are characterized by a “CpG island methylator phenotype” (CIMP), which classically refers to tumors with elevated levels of global DNA methylation rather than specific gene promoter hypermethylation status. Of note, it was recently demonstrated that CIMP is prevalent in melanoma patients with specific genetic alterations, including *NRAS*, *ARID2,* and *IDH1* mutations [44]. Particularly, *ARID2* and *IDH1* genes are involved in chromatin remodelling, which suggests a direct connection between somatic mutations and epigenetic dysfunction in melanoma. Similarly, the BRAF^V600E^ in cutaneous melanoma is associated with hypermethylation of suppressor gene promoters, while hypomethylation affects specific oncogene promoters, such as *FGD1* and *HMGB2*, which plays a direct role in both proliferation and inhibition of apoptosis [53,54]. A possible explanation is that the BRAF cascade may affect the expression of genes involved in maintaining the correct methylation status, such as the DNA methyltransferase (DNMT)-1, namely a negative regulator of DNA-methylation at CpG sites [54]. However, further mechanisms regulating the de-methylation of DNA remain to be elucidated. These speculations seem to be confirmed by recent discoveries obtained in murine models bearing the *BRAF* mutated colorectal cancer that proved that the persistent oncogenic BRAF signalling is sufficient to induce a progressive widespread DNA methylation [55].

Although data in melanoma are still limited, a previous observation in different cancer models revealed that alterations in the DNA methylation status also take part in the impaired tumor immunogenicity and immune recognition by inhibiting the expression of key molecules required for the interaction of cancer cells with the host’s immune system [56]. These epigenetic modifications may affect all phases of the antigen process, including the suppression of tumor-associated antigen expression and processing [57], the reduced synthesis of MHC class I molecules [58], and the impaired expression of accessory/co-stimulatory surface proteins [59]. Comprehensively, these data support the use of DNA hypomethylating agents (DHAs) in combination with immunotherapy to improve the antitumor activity, particularly in the context of poorly immunogenic tumors that still represent a major therapeutic challenge.

### 3.2. Histone Modifications

Eukaryotic cells package their DNA molecules in highly systematic chromatin structural units such as nucleosomes that are wrapped around an octamer of histone proteins organized in double sub-units, named H2A, H2B, H3, and H4. Histones can undergo post-transcriptional modification (PTMs), including acetylation and methylation of histone lysine residues that influence the chromatin structure. These kinds of ‘chromatin marks’ typically affect the N-terminal tails of histones and influence the nucleosomal stability and the accessibility of RNA-polymerases to DNA, which regulates gene transcription [60]. Globally, histone acetylation is associated with gene expression activation, whereas histone methylation correlates to either activation or repression of gene transcription. Given the important role of histone PTMs in epigenetics, it is not surprising that aberrant patterns of these chromatin marks are found in cancer.

In this context, the acetylation of the histone H3 protein at the 27th lysine residue (H3K27ac) was recently described as an important mechanism regulating the microphtalmia gene (MITF) expression, which confers an increased metastatic potential to melanoma cells [61]. Histone hypoacetylation is also involved in the progression of melanoma cells since the reversible deacetylation by histone deacetylases (HDAC) can down-regulate several onco-suppressor genes, such as *CDKN1A* and *phosphatidylinositol-4,5-biphosphate 5-phosphatase A (PIB5PA)* [62,63]. These observations lead to a pre-clinical evaluation of HDAC inhibitors in melanoma, which demonstrated a transient effect on arresting the cell cycle in G2/M phases through the accumulation of dephosphorylated retinoblastoma (RB) protein, which supports a potential application as anticancer treatment [64].

On the other hand, alterations of histone methylation may play a direct role in the transformation of melanocytes to melanoma cells. Histone methylation may occur on lysine and/or arginine residues, even though lysine methylation is most common and depends on stepwise addition of one-to-three methyl groups at various positions along the histone N-terminal tail [64]. This stepwise conversion from a not-methylated to a tri-methylated lysine residue is facilitated by histone methyltransferases (HMTs), while the reverse process known as demethylation is catalyzed by histone demethylases (HDTs). As largely described in previous studies, the defect of these enzymes in melanoma corresponds to histone methylation deregulation with consequent aberrant gene expression, including *SOX2*, *INK4b-ARF-INK4a*, and *ERK* [65]. In addition, different HDTs play a direct role in melanoma as well as in other tumor transformations while the hyper-activation of H3K4 and H3K9 demethylases may affect cell differentiation, senescence, and stemness potential [66,67,68]. Of note, two different types of H3K9 demethylases (LSD1 and JMJD2C) were found to disable the oncogenic MAPK-induced senescence by enabling the expression of several E2F target genes, such as the *CDK2*, *CCND2*, *CNE1*, and *DHFR* [68]. Lastly, more complex regulations of gene expression during melanomagenesis depend on histone variants, such as non-allelic isoforms of the conventional histones that are generally coded by distinct genes [69]. Apart from histone H4, the others include a number of variants, and those reported to correlate with melanoma progression are the histone H2A and the macroH2A (mH2A) as well as H2A.Z and H2A.Z.2 variants [70]. Comprehensively, aberrant expression of these histone variants in melanoma cells was correlated with the transcription of key genes implicated in the regulation of affecting cell-cycle dysregulation, increased migratory capacity, and metastatic propensity [71,72,73,74].

### 3.3. Chromatin Remodelling

Chromatin remodelling consists of the rearrangement of chromatin from a condensed state to a transcriptionally accessible one and depends on the activity of the polycomb group (PcG) proteins, which assemble in variable chromatin remodelling complexes. These complexes provide the mechanism for modifying chromatin by allowing transcription factors and other DNA binding proteins to access the DNA strand and control gene expression [75]. In this context, the abnormal function of polycomb repressive complex 2 (PRC2) was found to be associated with the initiation of a transcriptionally repressed state by the tri-methylation of H3 at lysine 27 (H3K27me3). Noteworthy, the enhancer of zeste homolog 2 (EZH2) is the core subunit of PRC2 endowed with methyltransferase activity, which is up-regulated in melanoma with respect to benign naevi [76]. The increased expression of EZH2 in melanoma results in the hyper-activation of PRC2 with consequent tri-methylation of H3K27, silencing of *CDKN2A* and *CDKN1A* tumor suppressor genes, and is associated with thicker primary melanomas, aggressive metastatic behavior, and a poor prognosis [77]. Noteworthy, the H3K27-mediated downregulation of *CDKN1A*, which is normally under the control of p53 protein, substantially contributes to evade the major mechanisms regulating cell cycle arrest and induction of a senescence phenotype [75]. In addition to EZH2 activation, other PcG proteins are frequently deregulated in melanoma, including the chromatin remodelling complex SWI/SNF [78] and the chromatin assembly factor-1 (CAF-1) [79]. The importance of chromatin remodelling deregulation in melanoma brought to investigate EZH2 inhibitors in EZH2-enriched tumors reveals promising pre-clinical activities [80].

### 3.4. Non-Coding RNAs

Non-Coding RNAs (ncRNAs) are classified into two groups, such as long (lncRNAs) and small (sncRNAs) that are differentiated in relation to the number of bps longer or smaller than 200. Small ncRNAs are further classified in micro- (miRNAs), piwi-interacting- (piRNAs), and small nucleolar-RNAs (snoRNAs). However, other ncRNAs in this group with less characterized activity have also been described [81]. In recent decades, the increasing interest in melanoma epigenetics has been particularly devoted to studying the deregulation of lncRNAs and miRNAs. The major mechanism involved with the epigenetic control exerted by lncRNAs depends on the recruitment of histone-modifying complexes (e.g., PRC) or other regulatory proteins at specific DNA target regions, which silences or activates gene promoters [82,83]. By contrast, miRNAs (19–22 bps) block the gene expression at the post-transcriptional level mostly by interfering with the 3′UTR region of mRNAs [84]. A class of lncRNAs has been recently described to exert a role as decoys or sequesters of miRNAs, while others also act by stabilizing the translational ribosomal machinery [10,85,86].

Several lncRNAs have been described and found to be deregulated in melanoma. The *BRAF-activated ncRNA* (*BANCR*) has been identified as a putative regulator of melanocyte transformation, which affects both cell proliferation and the migratory attitude by activating the ERK1/2 and MAPK pathways [87,88]. The *HOX transcript antisense RNA* (*HOTAIR*) is another lncRNA particularly enriched in melanoma metastasis, while being poorly expressed in primary lesions [89]. Its role in the pro-metastatic process has been widely investigated and it was demonstrated that *HOTAIR* interacts with the PRC2 favoring the tri-methylation of H3K27 at specific target genes with anti-metastatic activity. Thus, epigenetic processes silence their expression [90]. Another lncRNA (*SPRY4-IT1*) was identified as a regulator of both apoptosis and differentiation in melanoma [91], while further study in non-small cell lung cancer (NSCLC) also revealed a possible role in the activation of the EMT machinery by regulating both E-cadherin and vimentin expression [92]. Lastly, the *SAMMSON (survival associated mitochondrial melanoma-specific oncogenic lncRNA*) is considered one of the major lncRNA implicated in melanomagenesis, whose expression occurs in more than 90% of melanomas [93]. The most relevant activity of *SAMMSON* is the activation of the mitochondrial p32 protein, which is a critical regulator of tumor metabolism via maintenance of oxidative phosphorylation and mitochondrial homeostasis [94]. The role of this lncRNA in melanoma has promising therapeutic implications since the combination of inhibitors of both BRAF and SAMMSON in pre-clinical models apparently exert a synergistic anti-tumor effect [93].

The role of miRNAs in melanoma is very tangled and, therefore, we recently reviewed this topic in detail [95]. In particular, miRNAs influence the progression of melanoma and impair the immune system activity as well as prepare the formation of the pre-metastatic niche. An area of interest in this context is the biological relevance of melanoma-related miRNAs in the propagation of pro-tumoral signals, which affects the tumor microenvironment and immune system cells. In this regard, the cargo of miRNAs via melanoma-derived Exo has a pivotal role in tumor progression as described for miR-9, whose exosomal transfer into endothelial cells can activate their migratory and pro-angiogenic potential by prompting the signal transducer and activator of transcription 3 (STAT3) pathway [4,96]. A similar perturbation of the JAK/STAT pathway in melanoma cells depends on the exosomal transfer of miR-24-3p, miR-891a, miR-106a-5p, miR-20a-5p, and miR-1908, which co-participate in the generation of an immunosuppressive milieu by promoting the Th1-polarization of CD4^+^ cells as well as the expansion of T-regulatory cells (T-regs) and myeloid-derived suppressor cells (MDSCs) [97].

The altered miRNA profile of melanoma cells was also correlated with their metastatic potential. In this context, high expression of miR-214 was found to be implicated in cell motility and increased migratory propensity, which drives the spreading of melanoma cells from the primary lesion to metastatic sites through the modulation of adhesion molecules and metalloproteases [98]. The miR-200 family, moreover, is considered of particular relevance for the metastatic process of melanoma cells, since it drives the EMT process by negatively influencing the zinc finger E-box-binding homeobox 1 (ZEB1) and E-Cadherin, while stimulating the expression of N-Cadherin and vimentin [99].

A very interesting area of research is progressively exploring the possible connections between deregulation of ncRNAs in melanoma and their propagation toward distant cells as well as pre-metastatic organs. In this context, it is conceivable that EVs, and particularly Exo, may play a pivotal role since they are optimal vectors carrying proteins and miRNAs and were found to dynamically modify their “epigenetic” cargos in adaptation to different microenvironmental conditions or drug pressures [100].

In conclusion, it is conceivable that epigenetic modifications occur in melanoma and prompt the metastatic potential that stimulates a critical mechanism regulating the spreading of melanoma cells from primary to secondary sites. Therapeutic strategies aimed to restrain the epigenetic defect in combination with targeted agents and/or immunotherapy could be planned in future investigative clinical trials.

## 4. Clinical Applications of Extracellular Vesicles and Melanoma Epigenetics

The recent acquisitions in the field of EVs and epigenetics allowed the development of novel strategies with a potential translation in the clinical setting. The isolation and characterization of melanoma-derived EVs are an attractive tool for both diagnostic and prognostic purposes, while new therapeutic approaches include the use of epigenetic drugs and engineered nanocarriers loaded with ncRNAs.

(i) Circulating biomarkers. Both tumor-derived RNAs and DNAs are packaged within the phospholipidic bilayer of EVs and are protected from degradation by serum ribonucleases and DNases. Therefore, the analysis of exosomal miRNAs has been widely investigated as an innovative strategy for a liquid biopsy. The liquid biopsy is emerging as a helpful alternative to conventional tissue biopsy, which provides a non-invasive approach for the detection and real-time measuring of cancer biomarkers by simple drown of blood procedures as well as other biological fluids including urine, saliva, or ascites [101]. In this context, increased levels of exosomal miR-17, miR-19a, miR-21, miR-126, and miR-149 were measured in the plasma of patients with sporadic metastatic melanoma as compared to a healthy control and proposed as possible biomarkers in the clinical setting [102,103].

Moreover, exosomal DNA has been sequenced to detect the presence of *BRAF^V600^*, while high levels of Exo-miR-211 was correlated with reduced sensitivity to BRAF inhibitors in metastatic melanoma, which supports molecular analyses of Exo to predict the responsiveness to targeted agents [30,104]. A potential and very useful application of circulating Exo concerns the isolation of EVs originating from the immune cells. As we recently demonstrated, T-cell-derived Exo are a bona fide representation of the immune system health, which reflects the intrinsic propensity of the immune system to be reactivated by immune-checkpoint inhibitors [105].

(ii) Novel therapeutic strategies. Melanoma is a highly immunogenic tumor and immunotherapy becomes a backbone for the treatment of this cancer since a monoclonal antibody directed against the Cytotoxic T-Lymphocyte Antigen-4 (CTLA4) receptor demonstrated its effectiveness in a limited number of metastatic patients [106]. Other immune-checkpoint inhibitors and their combination have dramatically raised the percentage of patients that benefit from immunotherapies, even though alternative strategies are still needed to improve the efficacy of these drugs, especially in poorly immunogenic cancers.

In this context, epigenetic drugs including DHAs have been explored in pre-clinical models demonstrating their ability to potentiate immune recognition through the up-regulation of the human leukocyte antigen (HLA) class I and accessory/co-stimulatory molecules, the modulation of Th1 polarization, and the promotion of CD8^+^ T-cell activation and proliferation [58]. Despite previous approvals for the treatment of certain haematological malignancies, the efficacy of first-generation DHAs (e.g., azacytidine and decitabine) in patients with solid tumors has been disappointing [107]. Next-generation DHAs, however, demonstrated promising immune-modulatory and anti-tumor activity in clinical trials [56]. In this continuously evolving scenario, the phase 1b NIBIT-M4 study recently confirmed the tolerability and safety of guadecitabine associated with ipilimumab in unresectable stage III/IV melanoma, which shows a unique objective response rate of 26% [108].

Other innovative approaches aimed at restoring effective anti-tumor immune responses exploit Exo derived from dendritic cells (Dex) to induce an antigen-specific T-cell activation. Early phase clinical trials were designed to verify the potential use of autologous Dex pulsed with tumor peptides for the immunization of stage III/IV melanoma patients as cell-free anti-melanoma vaccines [109,110]. Despite these studies demonstrating the feasibility of large-scale production of Dex and the safety administration, further validation of their anti-tumor efficacy is still needed. The selective delivery of active drugs using nanovesicles is also a possible strategy. To this, Van Woensel et al. recently proposed anti-Galectin-1 siRNA-loaded chitosan-nanoparticles in mice with glioblastoma, which demonstrates increased CD4^+^ and CD8^+^ T-cell activation following their administration and potential anti-tumor activity [111]. The combination of these agents with other treatments aims to improve the efficacy of chemotherapy and immunotherapy, such as the administration with temozolomide and anti-PD1 immunotherapy. Finally, a major unmet issue in melanoma is targeting mutated forms of NRAS kinases, which are still considered undruggable. Thus, alternative strategies including siRNAs and artificial-miRNAs (amiRs) have been proposed for directly inhibiting the downstream transduction of specific *RAS* point-mutations [112,113]. Innovative nano-carriers are currently under investigation as a possible system for the selective delivery of these molecules into cancer cells [114,115].

## 5. Conclusions and Future Perspective

Recent acknowledgment in the molecular landscape of melanoma has brought impressive results in terms of overall survival in metastatic disease. However, the prognosis of a number of patients remains particularly poor and resistance to therapy is still a challenge. Recent studies, nonetheless, showed the relevant role of EVs and epigenetic deregulation in melanoma progression and opened new strategies for both diagnosis and treatment. Moreover, a high number of epigenetic modifications add further layers of complexity to this system and have been associated with peculiar biological aggressiveness or activation of specific pathways. In this context, DNA methylation as well as histones and chromatin remodeling are potentially druggable targets whose investigation is rapidly proceeding from pre-clinical studies into clinical trials. The potential interactions between melanoma epigenetics with both MAPK signaling and immune system activity require further efforts to better qualify the possible role of epigenetic drugs in overcoming drug resistance as well as potentiating the anti-cancer effects of new immune checkpoint inhibitors.

Many efforts in past years have been devoted to investigate tumor-derived Exo as circulating biomarkers or possible druggable targets, particularly due to their link with intercellular communication and epigenetic deregulation of cancer cells. However, although EVs seem very promising for their structural and pharmacological properties, their translation into the clinical arena seems far from a direct application. In addition, a previous study investigating a possible use as an anti-melanoma vaccine failed to demonstrate a real benefit by adopting this approach. Future directions include the development of intelligent nanoparticle systems for selective gene therapy approaches as innovative treatment modalities.
